# Portfolio effects and functional redundancy contribute to the maintenance of octocoral forests on Caribbean reefs

**DOI:** 10.1038/s41598-022-10478-4

**Published:** 2022-05-02

**Authors:** P. J. Edmunds, H. R. Lasker

**Affiliations:** 1 Department of Biology, California State University, 18111 Nordhoff Street, Northridge, CA 91330-8303 USA; 2 Department of Environment and Sustainability and Department of Geology, University at Buffalo, Buffalo, NY 14260 USA

**Keywords:** Ecology, Ecology, Environmental sciences, Ocean sciences

## Abstract

Declines in abundance of scleractinian corals on shallow Caribbean reefs have left many reefs dominated by forests of arborescent octocorals. The ecological mechanisms favoring their persistence require exploration. We quantified octocoral communities from 2014 to 2019 at two sites in St. John, US Virgin Islands, and evaluated their dynamics to assess whether portfolio effects might contribute to their resilience. Octocorals were identified to species, or species complexes, and their abundances and heights were measured, with height^2^ serving as a biomass proxy. Annual variation in abundance was asynchronous among species, except when they responded in similar ways to hurricanes in September 2017. Multivariate changes in octocoral communities, viewed in 2-dimensional ordinations, were similar between sites, but analyses based on density differed from those based on the biomass proxy. On the density scale, variation in the community composed of all octocoral species was indistinguishable from that quantified with subsets of 6–10 of the octocoral species at one of the two sites, identifying structural redundancy in the response of the community. Conservation of the relative colony size-frequency structure, combined with temporal changes in the species represented by the tallest colonies, suggests that portfolio effects and functional redundancy stabilize the vertical structure and canopy in these tropical octocoral forests.

## Introduction

Tropical coral reefs have undergone global declines in the percentage cover of scleractinian corals over the last four decades, and these declines have altered the relative abundances of coral species^1,2^. Species that produce long-lived, massive, and architecturally complex colonies have tended to decline in population size^3,4^, while corals with weedy life history strategies that produce small and structurally simple colonies have tended to increase in abundance^3,5^. These trends have been accompanied by changes in the community structure and function of coral reefs^3,6^, and while caused by multiple types of disturbance, most can be attributed to anthropogenic effects^2,7–9^. In the Caribbean, where coral mortality has been acute^10,11^, these losses have been accompanied by rising abundances of macroalgae^12,13^, and sometimes other benthic macro-invertebrates including octocorals, sponges, and ascidians^14–17^. For octocorals, persistent increases in population sizes suggests that octocoral forests could represent a new “normal” for shallow benthic communities (sensu Lasker et al.^18^), but this prediction depends on the extent to which they are ecologically resilient on present-day reefs.

Damage through disturbance and subsequent recovery is an integral component of the ecological and evolutionary history of coral reefs^19,20^, but their future persistence will depend on their resilience to disturbances differing in frequency, intensity, and type, relative to the past^9,21^. The changes affecting Caribbean reefs have resulted in octocorals increasing in abundance over the last 25 years^16,22,23^, but this long-term trend includes multi-year dynamics over which abundances have waxed and waned^16,18,24^. Species-level data for octocorals have been collected by a small number of taxonomically skilled researchers^18,23,25,26^, but like many analyses of benthic communities on coral reefs^10,27^, most studies of this taxon have focused on abundances pooled among species^22,28^. Taxonomic pooling prevents holistic analyses of community dynamics, or consideration of properties arising from assemblages of species or genera such as the relationship between diversity and stability^29^, functional redundancy^30^, portfolio effects^31^, response diversity^32^, and the demographic underpinnings of community structure^33^. For the diverse fauna supporting the increases in octocoral abundances on Caribbean reefs^18,25,34^, it is timely to evaluate the roles played by individual species in forming animal forests^35,36^ and in mediating the response of these forests to disturbances.

Studies of portfolio effects and functional redundancy have become common in analyses of community resilience^31,37–39^. Much of the attention has considered whether these concepts can identify communities as candidates for conservation based on their high resilience^38,40^, or determine whether the processes mediating resilience can be manipulated to enhance resilience^41,42^. The presence of portfolio effects is inferred when the species making up a community exhibit heterogeneous responses to common conditions with little change in aggregate community properties^39^, and thus portfolio effects are similar to the concept of response diversity^32^. Functional redundancy is more elusive, because it requires an understanding of how traits affect, or are correlated with, ecosystem function^32,43^. This issue does not have a simple solution, since a species’ effect on ecosystem scale functions is itself a function of a suite of traits, most of which are rarely codified, quantified, or evaluated for impacts on community function. Bellwood et al.^32^ recently addressed this issue, highlighting ambiguity over the use of ‘function’ in ecology, and advocating for a meaning focusing on the movement or storage of energy or material. They also suggest distinguishing between functions defined by phenotype (i.e., “how” based) versus ecosystem (i.e., “what” based). Independent of the trait(s) for which functional redundancy is considered, this property cannot be demonstrated without manipulative experiments^30^.

This study focuses on the octocoral forests of St. John and evaluates the relationships between community resilience and changes in abundances and sizes of arborescent colonies^18,43^. Using phenotype as a proxy for function (sensu^32^), we hypothesized that multiple species perform similar functions, with these functions related to their arborescent morphology and their ability to form canopies through vertical structure. If this hypothesis is correct, a reduction in abundance of one species might be compensated for by the persistence, or change in abundance, of other species performing similar functions. Community resilience might, therefore, be enhanced by complementary population dynamics in response to disturbance, changing environmental conditions, or stochastic fluctuations in population abundance; such dynamics commonly are described as portfolio effects^31,37,38^. Functional redundancy^30^ can be considered within this framework, and it describes complementary dynamics arising from multiple species performing similar functions that can be exchanged without affecting community properties such as ecological resilience.

Using a mensurative approach^44^, we describe octocoral communities from 2014 to 2019 at two sites on the south coast of St. John and evaluate whether variation in species abundances are consistent with portfolio effects. The reefs at these sites are exposed to contrasting environmental conditions (waves, light, and sedimentation), and while their octocoral communities have nearly identical species richness, they differ in colony density, colony heights, and relative species abundances^18,45^. The study included the effects of two category 5 hurricanes in 2017^46,47^, which reduced octocoral densities without greatly altering community structure^18^. We identified octocorals to species and measured abundances (colonies m^−2^) and colony height. The square of colony height (height^2^) was used as a proxy for biomass (hereafter, “biomass proxy”), and colony size-frequency structure was used to characterize the physical structure of the animal forest created by the octocoral colonies. Colony size-frequency patterns together with colony density were used to evaluate how the emergent functional property of the community’s vertical structure changed over time, and whether there was evidence of portfolio effects and functional redundancy for the vertical structure of the octocoral forest.

First, we describe univariate changes in populations using density and the biomass proxy. Second, we quantify community synchrony (ϕ [after Thibaut and Connolly^37^]) and compare octocoral forests against other systems with respect to the relative importance of portfolio effects. Third, we describe multivariate abundance using 2-dimensional ordination, and use the statistical property, “structural redundancy”^48^, to determine whether subsets of species create ordinations that are indistinguishable from the ordination obtained with the full taxonomic complement. Where structural redundancy is detected, and the interchangeable species are functional equivalents, this approach can be interpreted as addressing functional compensation^49,50^ as a mechanism modulating assemblage resilience^48^. Functional compensation (or complementarity^50^) is similar to functional redundancy but focuses on the maintenance of community function (not the interchange of species function) through species exchange^50^. Finally, we tested for variation in the way in which octocorals utilized the three-dimensional volume above the benthos through changes in the frequency distribution of colony heights, as well as the species contributing colonies to specific size classes. These approaches indirectly evaluated the distribution of octocoral biomass perpendicular to the benthos and its relationship to octocoral community composition.

## Methods

### Descriptive ecology

Octocorals were surveyed on the fringing reef along the south shore of St. John, at Grootpan Bay (18.310° N, 64.719° W) and Europa Bay (18.317° N, 64.730° W). The study sites and experimental design are described in our early papers^24,51^, but note we have changed the name of East Cabritte to Grootpan Bay to enhance geographic accuracy.

At each sampling, the identity and height of octocoral colonies ≥ 5-cm tall were recorded in the study plots, with analyses focusing on arborescent species. *Erythropodium caribaeorum* and encrusting forms of *Briareum asbestinum* were not included. Colonies were identified to species during the surveys. In cases of uncertainty, voucher specimens (branches fragments < 5 cm long) were collected for sclerite analyses in the lab (after Bayer^34^). *Eunicea laxispica, E. mammosa, E. succinea* as well as *Pseudoplexaura flagellosa and P. wagenaari* were difficult to resolve, and to minimize the number of colonies sampled, these species were summed by genus and reported as *E. mammosa* complex and *P. flagellosa/wagenaari*. Analyses of sclerites confirmed that all 5 species were present at the study sites. Variation over time in the octocoral communities at each site was described in a univariate framework, first using density (colonies m^-2^), and second, the sum of height^2^, which we use as a biomass proxy. Our biomass proxy was rationalized by the quadratic relationship between height and biomass that characterizes *Antillogorgia elisabethae* in the Caribbean^52^, as well as two Pacific *Muricea* spp.^53,54^. While the relationship between height and biomass is likely to differ between species, arborescent octocorals expand laterally as colonies increase in height, and both the number of branches and the volume of seawater occupied by branches is correlated with colony height. Results are presented for total abundance (i.e., pooled among taxa), and for the three most common species on density and biomass proxy scales.

### Portfolio effects

To test for portfolio effects, we used the approach of Thibaut and Connolly^37^ and quantified community synchrony (Φ), the extent to which taxa respond in similar ways to changing conditions, and community variability (${CV}_{n}^{c}$), the inverse of stability and a measure of the extent to which communities vary over time. Species abundances averaged by sampling period and site were used to calculate Φ and ${CV}_{n}^{c}$. Φ is the ratio of empirical community variance between surveys and the variance expected if all the taxa changed in complete synchrony. ${CV}_{n}^{c}$ is the product of the square root of Φ and the mean species-level coefficient of variation for the whole community between surveys (Eqs. 2–4 in Thibaut and Connolly^37^).

### Structural redundancy

Multivariate community structure at each site was tested for structural redundancy^48^, based on resemblance matrices and 2-dimensional ordinations prepared using non-metric multidimensional scaling (hereafter, MDS). In this approach, the resemblance matrix based on the assemblage of common species is tested for multivariate, non-parametric correlation (with ρ as the test statistic) with a second resemblance matrix prepared using a subset of species drawn from the assemblage of common species using a forward selection/backward elimination algorithm^48^. The analysis was restricted to common species by removing rare taxa that together accounted for ≤ 2% of all octocorals^48^. Where the resemblance matrices of the subset versus the full set are statistically indistinguishable (*P* > 0.05), the species subset is subtracted from the full species assemblage and the process of removing species is sequentially repeated until the resemblance matrix significantly differs from that of the full species assemblage. These analyses identify subsets of taxa, “peels”, whose variation in abundances match that of the overall community. The number of significant “peels” of the full species assemblage measures structural redundancy in the response of the taxa, where select members of each peel are inferred to be structurally redundant with select members of other peels, or the full species set.

Having excluded rare taxa, abundances by species were log(x + 1) transformed and expressed as resemblance matrices using Bray Curtis dissimilarities. Ordinations by MDS were prepared using 100 restarts or until stress stabilized at < 0.1, and were plotted with symbols scaled to overall octocoral abundance (colonies m^−2^). Tests for structural redundancy were completed using a stepwise function (BVSTEP) in Primer 6 software^55^, with multivariate correlations tested in a permutational framework (999 permutations). Analyses with sequential removal of species subsets were completed until resemblance matrices differed from the matrix prepared with the full species assemblage.

### Colony size structure of the octocoral forests

The physical structure of the octocoral forests was characterized as colony size frequency distributions, both pooled among species and considered separately for the most common species. Size frequency distributions (pooled among species) were compared between sites and time using log-linear analyses (with SPSS v 21 software) using five size classes (I < 10 cm, II ≥ 10 and < 20 cm, III ≥ 10 and < 40 cm, IV ≥ 40 and < 60, and V ≥ 60 cm), and differences in the distributions were visualized using MDS (with Primer 6 software). The shapes of the distributions were also characterized using adaptations of the species diversity indices H' and Evenness, where the height classes were treated as analogues of species and numbers of colonies in each size classes as analogues of species abundances. To evaluate the role of individual species in supporting each colony size class, the contribution to each colony size class of the ten most common species (with the remainder pooled as “others”) at each site and sampling point was expressed on a proportional scale and displayed using fill plots. For clarity, these plots were prepared using four size classes (< 20 cm, ≥ 20 and < 40 cm, ≥ 40 cm and < 60 cm, and ≥ 60 cm).

## Results

### Descriptive ecology

**Figure 1:**
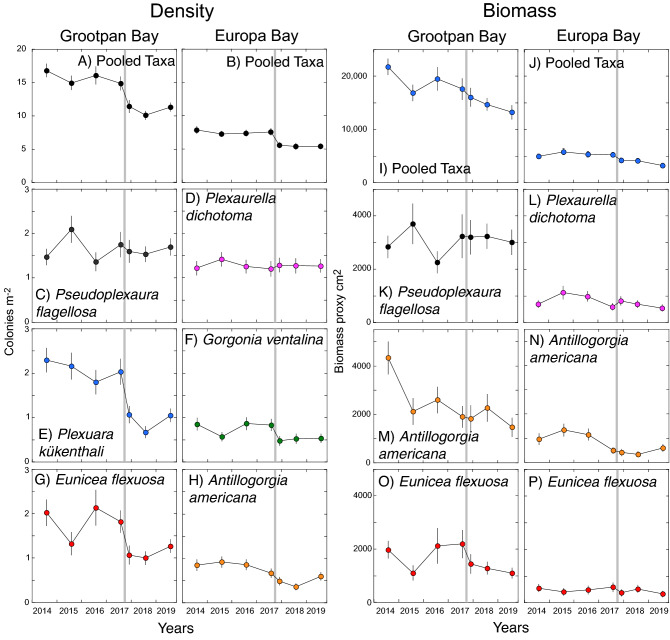
Octocoral community structure (density, colonies m^−2^, and biomass proxy, cm^2^) at Grootpan Bay and Europa Bay from 2014 to 2019. Values show mean ± SE (n = 28–60 quadrats) for pooled taxa (**A**, **B** and **I**, **J**) and the three most common taxa by density (**C**–**H**) and the biomass proxy (**K**–**P**). Gray bars show the impact of Hurricanes Irma and Maria.

Over the study, 7 surveys were completed, and 41 taxa of octocorals were identified at Grootpan Bay, and 39 at Europa Bay. Mean densities (± SE, pooled among taxa) varied from 10.1 ± 0.7 colonies m^−2^ (2018) to 16.8 ± 1.0 colonies m^−2^ (2014) at Grootpan Bay, and from 5.4 ± 0.5 colonies m^−2^ (2018) to 7.9 ± 0.5 colonies m^−2^ at Europa Bay (2014). At Grootpan Bay, mean densities declined 23% from July 2017 to November 2017 in association with Hurricanes Irma and Maria, and at Europa Bay they declined 26% (Fig. 1A,B). By density, the three most common taxa at Grootpan Bay accounted for 35% of colonies averaged across years, and included *Pseudoplexaura flagellosa/wagenaari* (12.0% of colonies), *Plexaura kükenthali* (11.6%), and *Eunicea flexuosa* (11.1%). At Europa Bay, the three most common species accounted for 39% of colonies averaged across years, and included *Plexaurella dichotoma* (19.1%), *Antillogorgia americana* (10.2%), and *Gorgonia ventalina* (10.0%) (Fig. 1C–H). Densities of these six species changed heterogeneously. At Grootpan Bay, the density of *P. flagellosa/wagenaari* was unaffected by Hurricanes Irma and Maria, but the densities of both *P. kükenthali* and *E. flexuosa* declined in association with the storms (loss of 48% and 41% of colonies, respectively, Fig. 1C,E,G). At Europa Bay, *P. dichotoma* was unaffected by the hurricanes, but both *G. ventalina* and *A. americana* declined in density in association with the storms (43% and 27%).

Variation in the biomass proxy (i.e., height^2^) for octocorals over time at both sites was similar to that for density. At Grootpan Bay, the biomass proxy (± SE) for octocorals varied from a low value of 1326 ± 1307 cm^2^ (2019) to a high value or 21,740 ± 1506 cm^2^ (2014), and at Europa Bay, from a low value of 3260 ± 430 cm^2^ (2019) to a high value of 5830 ± 583 cm^2^ (2015). At Grootpan Bay, the mean biomass proxy trended downward over the study, and declined 9% from July 2017 to November 2017. At Europa Bay, the biomass proxy showed little variation, but declined by 19% in association with Hurricanes Irma and Maria (Fig. 1I,J). The three most common species at each site by biomass—*P. flagellosa/wagenaari*, *A. americana*, and *E. flexuosa* at Grootpan Bay, and *P. dichotoma, A. americana*, and *E. flexuosa* at Europa Bay—showed heterogeneous dynamics for the biomass proxy. At Grootpan Bay, neither the biomass proxy for *P. flagellosa/wagenaari* nor *A. americana were* affected by Hurricanes Irma and Maria, but the biomass proxy for *A. americana* showed a strong and near-linear decline over the study, and for *E. flexuosa* it declined by 34% in association with the hurricanes. At Europa Bay, the biomass proxy for *P. dichotoma* increased by 37% in November 2017 compared to July 2017, for *A. americana* it showed a downward trend over the whole study, and for *E. flexuosa* it was relatively stable, but declined by 37% in association with the hurricanes (Fig. 1K–P).

### Portfolio effects

**Figure 2:**
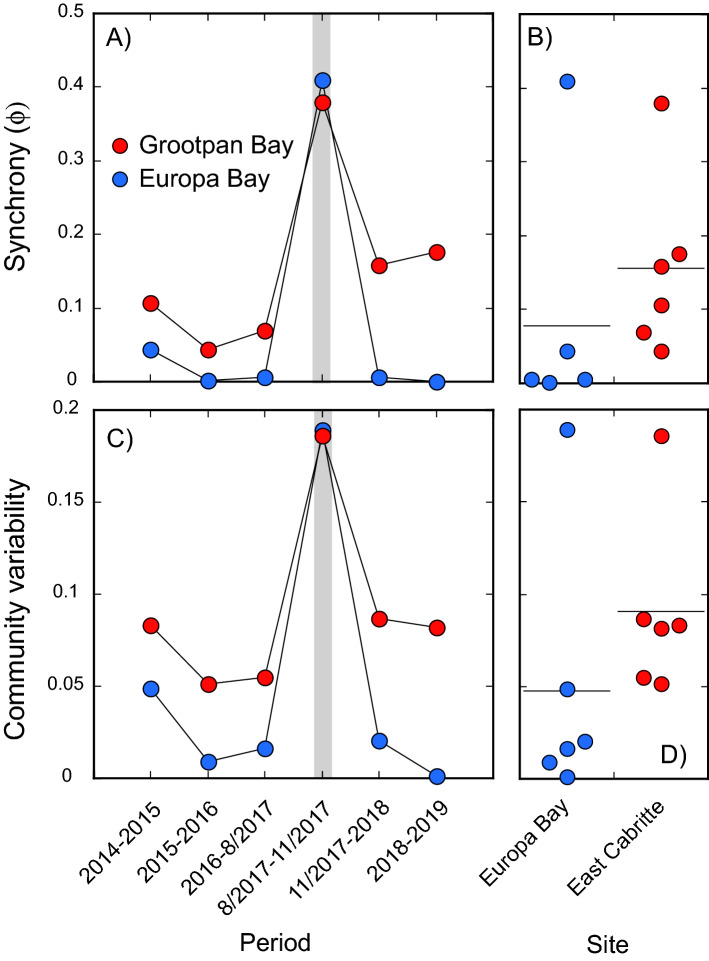
Community synchrony (ϕ) (**A**, **B**) and variability (${CV}_{n}^{c}$) (**C**, **D**) (after Thibaut and Connolly 2013) for Grootpan Bay (n = 41 species) and Europa Bay (n = 39 species) based on densities by species from 2014 to 2019. Line plots (**A**, **C**) show variation in ϕ and ${CV}_{n}^{c}$ over time, with Hurricanes Irma and Maria shown with the grey bar. Dot plots (**B**, **D**, showing means with horizontal line) display variation pooled over time for ϕ and ${CV}_{n}^{c}$.

Community synchrony (Φ) by species varied among surveys (Fig. 2A), ranging from 0.044 (2015–2016) to 0.379 (July 2017–November 2017) at Grootpan Bay, and from 0.002 to 0.410 at Europa Bay. The greatest synchrony was coincident with Hurricanes Irma and Maria (i.e., from July 2017 to November 2017); synchrony did not differ between sites (U = 7.000, P = 0.078) (Fig. 2A,B) in part due to the high synchrony associated with the hurricane effects at both sites. Community variability (${CV}_{n}^{c}$) also differed among surveys at both sites (Fig. 2C), with the relative variation similar to that of Φ. At Grootpan Bay, ${CV}_{n}^{c}$ varied from 0.052 (2015–2016) to 0.186 (July 2017–November 2017), and at Europa Bay from 0.009 (2015–2016) to 0.190 (July 2017–November 2017); ${CV}_{n}^{c}$ did not differ between sites (U = 6.000, *P* = 0.055) (Fig. 2C, D). Φ and ${CV}_{n}^{c}$ were strongly and positively correlated at both sites (r ≥ 0.977, df = 4, *p* ≤ 0.001).

### Structural redundancy

**Figure 3:**
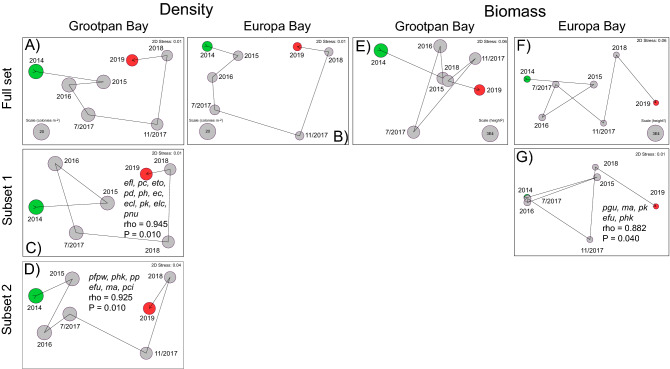
Two-dimensional ordinations by MDS of octocoral community structure by density (colonies m^−2^) and the biomass proxy (height^2^) for Grootpan Bay and Europa Bay. Ordinations shown for the full species assemblage (**A**, **B**, **E**, **F**) and for the first subsets of species that generate statistically indistinguishable ordinations. Species codes and full analyses in Table 1. No species subsets produced similar ordinations for density at Europa Bay, or for the biomass proxy at Grootpan Bay.

The MDS plots of the communities using colony densities were distinctive and repeatable (i.e., stress < 0.01) (Fig. 3A, B). At Grootpan Bay, incremental change across years caused the communities to become more dissimilar from 2014 to 2018, but more similar to the original state in 2019. The biggest change in the community occurred between November 2017 and 2018, and not between the samplings before and after the hurricanes in 2017 (Fig. 3A). At Europa Bay, the multivariate changes were broadly similar to those at Grootpan Bay, with divergence from 2014 to November 2017, and convergence to the original state from 2018–2019 (Fig. 3B). The greatest change in community structure was coincident with the hurricanes (i.e., from July 2017 to November 2017), but this barely differed from that occurring between November 2017 and 2018.

At Grootpan Bay the pattern of variation in multivariate community structure based on density was reproduced by two subsets of 6–10 species (Fig. 3C,D, Table 1). The first subset consisted of 10 species representing four genera, with *Eunicea* the most speciose (five species) in the subset. The second subset consisted of 6 species representing five genera, with *Pseudoplexaura* the most speciose (two species). These results identify structural redundancy on the density scale at Grootpan Bay. At Europa Bay, the ordination created with all species (Fig. 3B, Table 1) could not be recreated by any species subset and, therefore, there was no structural redundancy on the density scale at Europa Bay.

**Table 1:** Summary of octocoral species that generate ordinations by density and the biomass proxy that do not significantly differ from that produced by all taxa (Fig. 3) for Grootpan Bay and Europa Bay.

	Density		Biomass proxy	
	Grootpan bay	Europa bay	Grootpan bay	Europa bay
Subset 1	efl = *Eunicea flexuosa* pc = *Pseudoplexaura crucis* eto = *Eunicea tourneforti* pd = *Plexaurella dichotoma* ph = *Plexaura homomalla* ec = *Eunicea calyculata* ecl = *Eunicea clavigera* pk = *Plexaura kuna* elc = *Eunicea lacineata* pnu = *Plexaurella nutans*	NS	NS	pgu = *Pterogorgia guadalupensis* ma = *Muricea atlantica* pk = *Plexaura kuna* efu = *Eunicea fusca* phk = *Plexaura kükenthali*
	*ρ* = *0.945*	*ρ* = *0.826*	*ρ* = *0.796*	*ρ* = *0.882*
	*P* = *0.010*	*P* = *0.100*	*P* = *0.310*	*P* = *0.040*
Subset 2	pfpw = *Pseudoplexaura wagenaari* phk = *Plexaura kükenthali* pp = *Pseudoplexaura porosa* efu = *Eunicea fusca* ma = *Muricea atlantica* pci = *Pterogorgia citrina*			NS
	*ρ* = *0.925*			*ρ* = *0.805*
	*P* = *0.010*			*P* = *0.090*
Subset 3	*ρ* = *0.673*			
	*P* = *0.060*			

Taxa were filtered to remove the least common members that together account for < 2% of organisms (on density and biomass scales).

Multivariate variation in octocoral community structure by the biomass proxy differed from that described by density at both sites (Fig. 3E,F vs. 3A,B). On this scale, octocoral communities diverged from their 2014 state over the study at both sites (i.e., the community in 2019 was more dissimilar to that in 2014 than at any other time), and the changes coinciding with the hurricanes were similar to those occurring between 2016 and July 2017 at Grootpan Bay, and between August 2017 and 2018, and also from 2018 to 2019 at Europa Bay. No subset of species reproduced the temporal variation of the whole species pool at Grootpan Bay, but there was one species subset that could reproduce the whole species pattern at Europa Bay. This subset consisted of 5 species from 4 genera (Fig. 3G and Table 1), with no additional species subsets capable of reproducing the ordination from the full species assemblage. Overall, there was no support for structural redundancy on the biomass proxy scale at either Grootpan or Europa Bay.

### Size structure of the octocoral forests

**Figure 4:**
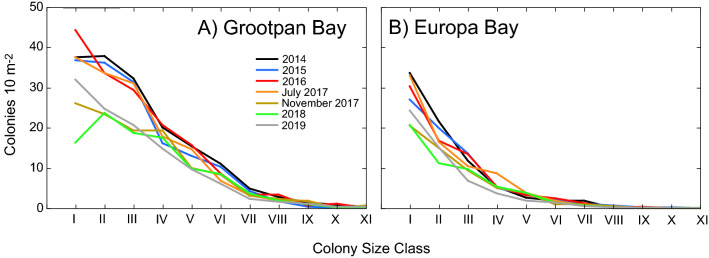
Size-frequency distributions of octocorals at Grootpan Bay (**A**) and Europa Bay (**B**), assessed annually in July of each year as well as November 2017. Frequencies are reported as the number of colonies in 10 m^2^, pooled among taxa. Size Class I = < 10 cm, II: ≥ 10 cm and < 20 cm, III: ≥ 20 cm and < 30 cm, IV: ≥ 30 cm and < 40 cm. V: ≥ 40 cm and < 50 cm, VI: ≥ 50 cm and < 60 cm, VII: ≥ 60 cm and < 70 cm, VIII: ≥ 70 cm and < 80 cm, IX: ≥ 80 cm and < 90 cm.

The size-frequency distributions of colonies (pooled among taxa) differed between sites (Fig. 4), and abundances declined with colony size. Log-linear analysis showed that the size-frequency distributions differed between sites (Table 2, Height × Site interaction, *P* < 0.001). Although there was a drop in abundance of smaller colonies following the hurricanes (Fig. 4), overall the size-frequency distributions did not differ among times as revealed by the absence of second and third order interactions involving Year × Height and Site × Year × Height (Table 2). Colony height diversity and evenness (Supplementary Information Fig. 1) provide holistic indices describing the size frequency distributions, and neither H' nor J' changed over time.

**Table 2:** Results of Log-linear analyses of colony size frequency distributions of octocorals among samplings (7 samplings in 6 years) and sites (Grootpan Bay and Europa Bay).

**K-way and higher-order effects**					
K	df	Likelihood ratio χ^2^	P	Pearson χ^2^	*P*
1	11	557.903	< 0.001	576.578	< 0.001
2	34	82.012	< 0.001	79.188	< 0.001
3	24	6.348	1.000	6.350	1.000

**Partial associations**					
Effect	df	Partial χ^2^	*P*		
Site × Year	6	1.426	0.964		
Site × Height	4	68.078	< 0.001		
Year × Height	24	14.258	0.941		
Site	1	172.992	< 0.001		
Year	6	41.097	< 0.001		
Height	4	343.814	< 0.001		

First order effects of site, year, and height (size classes: I < 10 cm, II ≥ 10 and < 20 cm, III ≥ 20 and < 40 cm, IV ≥ 40 and < 60, and V ≥ 60 cm) indicate that the number of colonies differed between sites, between years, and in the different size classes. The 3-way effect in the upper section is the Site x Year x Height interaction.

**Figure 5:**
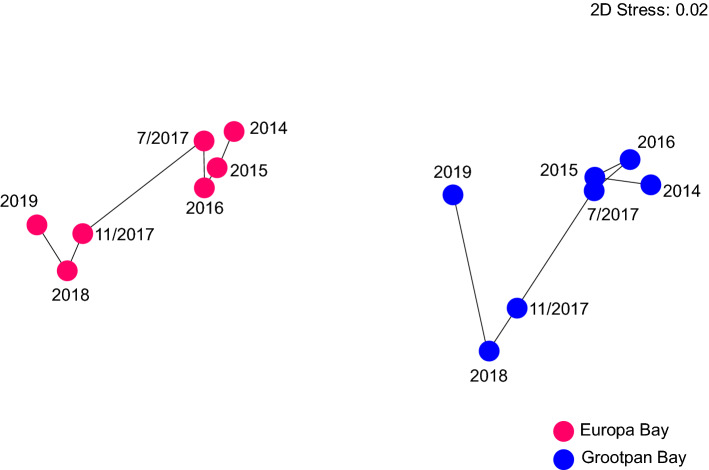
MDS ordination based on colony size frequency structure at Grootpan Bay and Europa Bay over seven samplings between 2014 and 2019. Separation along the abscissa is most strongly determined by membership of the four largest size classes (i.e., colonies ≥ 10 cm in height, II-V), whereas separation along the ordinate is most strongly determined by membership of the < 10 cm size class (I). Data were square root transformed prior to analyses, and the resemblance matrix was prepared using Bray Curtis dissimilarities.

Qualitatively, the extent to which the size-frequency distributions differed between sites and among times is displayed in MDS ordination space based on similarities in size class membership (Fig. 5). Grootpan Bay and Europa Bay separated in ordination space (i.e., they were distinct), but differences among years were similar between sites. At both sites, the size-frequency distributions in annual samplings from 2014 to July 2017 closely clustered in ordination space, showing that the distributions were similar over these periods. They substantially change from July 2017 to November 2017, with the post-hurricane differences persisting in 2018. There was a trend at Grootpan Bay for the size-frequency distribution to return to the pre-hurricane distribution. The abundances of colonies in Size Classes II-V were positively correlated with Dimension 1 in the MDS (r ≥ 0.87, df = 5, *P* ≤ 0.011), whereas Dimension 2 was positively correlated with the abundance of colonies in Size Class I (r = 0.88, df = 5, *P* = 0.009). The difference in size-frequency distributions in the MDS from Nov 2017 and 2018 versus the other years reflected the decline in abundance of all size classes, especially the decline of Size Class I at Grootpan Bay. The trend for recovery of the size-frequency structure in 2019 (relative to the pre-storm structure) was primarily driven by the increased abundance of Size Class I corals.

**Figure 6:**
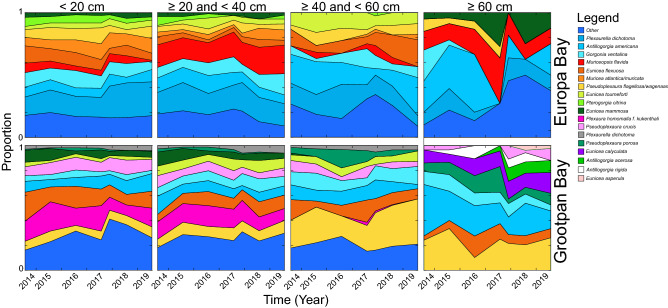
Proportional representation of the 10 most common octocoral species in four size classes at Europa Bay and Grootpan Bay in the 7 samplings described in this study. For Europa Bay, the most common 10 species were the same in each size class, but they varied among size classes for Grootpan Bay. Abscissa is plotted by sampling month, with two samplings in 2017, and axis marked in years. Sample sizes (number of colonies) by sampling at Europa Bay were: n = 176–266 for < 20 cm, n = 97–155 for ≥ 20 cm and < 40 cm, n = 23–52 for ≥ 40 cm and < 60 cm, and n = 8–23 for ≥ 60 cm; at Grootpan Bay: n = 112–261 for < 20 cm, n = 116–248 for ≥ 20 cm and < 40 cm, n = 67–146 for ≥ 40 cm and < 60 cm, and n = 24–54 for ≥ 60 cm.

While overall size frequency distributions did not change markedly over the study (Figs. 4 and 5), the relative abundances of the most common species within each size class varied over time and among size classes (Fig. 6). These changes are most evident in the larger size classes (i.e., ≥ 40 cm and < 60 cm, and ≥ 60 cm) where, for example, decreased abundances of *Antillogorgia americana* were matched at both sites by increased proportional representation of other taxa.

## Discussion

### Overview

Given the extent to which Caribbean coral reefs have changed in the last half-century^10,56,57^, as well as changes to the physical (e.g., temperature) and chemical (e.g., pH) conditions of the marine environment^58,59^, it is likely that the current success of octocorals is based on multiple aspects of their organismal biology. Presumably, the mortality of scleractinians^10,56^, the reduction in topographic complexity of reef surfaces^6^, and the provision of vacant space, which often has been exploited by macroalgae^13^, has also modified the coral reef habitat to the benefit of octocorals. However, the long-term success of octocorals on present-day reefs will depend on the ways by which these assemblages respond to current and future conditions. We have previously described how Hurricanes Irma and Maria in 2017 reduced the local abundances of octocorals in St. John without substantially affecting octocoral community structure and, thereafter, how these communities were recovering within two years of the disturbances through high recruitment^51^. Here, we have further explored patterns of variation in octocoral community structure over a slightly longer period and in greater detail, specifically to evaluate whether portfolio effects and functional redundancy could have played a role in mediating the dynamics we recorded. Our analyses focused on the roles of multiple species of arborescent octocorals in provisioning the physical structure of octocoral forests through the vertical elevation of colonies and the capacity of their distal branches to form a swaying canopy.

### Descriptive ecology

The present analysis expands on our previous work^18,45,51^ by adding an additional year to the time series describing the response of octocoral communities to Hurricanes Irma and Maria in September 2017^51^. The records of octocoral density over six years underscore the ecological resilience of the animal forests they produce (^18,51^ and present study), but colony density alone does not characterize octocoral community dynamics^60,61^. Our description of the octocoral community using density, a biomass proxy, and size-frequency distributions of colony heights, provides a holistic description of the emergent properties of octocoral forests, in particular their ability to contribute vertical structure and the resultant canopy and understory habitat^36,51,62^.

On St. John, height^2^, the biomass proxy, was positively correlated with colony density at Grootpan Bay (r = 0.892, df = 5, *P* = 0.007) and Europa Bay (r = 0.845, df = 5, *P* = 0.017), largely because the colony size-frequency distributions (pooled among taxa) were conserved over time, and the changes following Hurricanes Irma and Maria were limited to smaller colonies (Fig. 4), which had a small effect on the biomass proxy (height^2^). However, the colony size-frequency distributions differed between sites, and the apparent contribution of species to the community varied depending on whether density or the biomass proxy was used as a state variable. At Grootpan Bay the three most abundant species by density were *Pseudoplexaura flagellosa/wagenaari*, *Plexaura kükenthali* and *Eunicea flexuosa,* but by biomass, they were *P. flagellosa/wagenaari, Antillogorgia americana* and *E. flexuosa.* By identifying different species as major contributors to community structure depending on the state variable used for this task (i.e., density versus biomass proxy), our results highlight the importance of quantifying octocoral abundances using state variables other than density.

Octocoral forests in St. John have exhibited resilience over the six years of our study^43,51^. A theme in many analyses of community stability and resilience has been the stabilizing effects of functional redundancy, response variability, and portfolio effects^31^. The present analyses provides support for the role of these effects in maintaining species abundances, as well as the physical structure of the octocoral forest, on the shallow reefs of St. John. The low values of our metric of community variability (i.e., ${CV}_{n}^{c}$ ^37^) in most years demonstrate community stability, but community synchrony (Φ) at both sites remained relatively low (≤ 0.41, Fig. 1), with the exception of the interval including Hurricanes Irma and Maria. Φ quantifies the variance among species in their response to environmental conditions, and low synchrony in responses is consistent with the variation expected from portfolio effects^37^. Our results show that with the exception of the extreme event of two category 5 hurricanes in 2017, octocoral species in St. John responded in dissimilar ways to the common conditions prevailing from 2014 to 2019. Although synchrony in species abundances was high in the interval including the hurricanes, the effects on different size classes were more varied, suggesting a greater diversity of responses than indicated by population size alone. The temporal heterogeneity in octocoral dynamics that was revealed through Φ is not unusual relative to studies in which the same approach has been employed. For instance, the present values of Φ are within the range reported for grassland communities (i.e., low values of ca. 0.02–0.16^63–66^), although a meta analysis of 62 herbaceous plant communities revealed Φ values that mostly were > 0.4^67^. The asynchronous responses of octocorals in St. John are illustrated by the range of abundance patterns over time at our sites. For instance, *P. flexuosa/wagenaari* changed little in abundance over time; *P. kükenthali* sharply declined in abundance, and *E. flexuosa* displayed oscillatory dynamics. This diversity of responses to common conditions was displayed by octocorals at both sites and on both state variable scales (i.e., density and biomass proxy).

Functional redundancy describes cases in which species are ecologically equivalent in providing the same ecological function in terms of community services^68^. As a result, theory predicts that such communities will be resilient to disturbances acting in a spatio-temporal mosaic that impairs some (but not) all species at any one time^68^. A recent meta-analysis supports this assertion^39^. Structural redundancy, which we examined, identifies suites of species whose abundances are well correlated with that of the total community. The presence of multiple subgroups of species (i.e., “peels” sensu^48^) identified groups of taxa that are statistically similar to one another with respect to descriptions of multivariate changes in community structure. Where structural redundancy can be matched with taxon-specific phenotypes that support the notion of functional equivalency among them, structural redundancy can be interpreted as a measure of assemblage resilience and functional compensation^48^. As we describe below, much of the functional biology of octocorals remains to be quantified and, therefore, our ability to interpret the structural redundancy at Grootpan Bay is limited. Nevertheless, we posit that the presence of two structurally redundant subsets of species at Grootpan Bay is at least consistent with the notion of functional redundancy at the level of colony density. Classically, functional redundancy has been illustrated by the interdependence of the flow of energy through a species assemblage on the identity of the species in that assemblage^30^. A key challenge of this theory, however, is identifying the trait(s) for which redundancy is sought^43,69^. In the case of octocorals, colony abundances probably can be related to community-scale features such as productivity, but a comprehensive consideration of functional redundancy among tropical octocorals will require data not yet available for most species.

While functional redundancy is best informed by redundancy of the fundamental niche^69^, it often is evaluated from the perspective of a single, or a few, trait(s) considered to have ecological importance^32^. Colony morphology is an important component of the manner in which octocorals interact with their environment. Arguably, the most promising morphological traits to consider with respect to functional redundancy in arborescent octocorals are those associated with their vertical structure. The height of colonies, as well as the vertical distribution of their biomass in branches and polyps, will affect resource acquisition, interactions with other octocorals^70^, and their capacity to form canopies^18^. In terrestrial communities, the sizes and shapes of plants are important predictors of the outcomes of interactions among individuals^71,72^ and, more generally, the canopy created by assemblages of arborescent organisms can be considered an emergent property of dense stands of organisms (i.e., forests)^35^. The canopy modulates physical environmental features around the constituent organisms, thereby determining conditions within the understory habitat^51,62,73^, and the capacity of the forest to resist the destructive forces of the medium within which it operates (i.e., winds or the flow of water). While species identity in these systems undoubtedly mediates community properties, many of the emergent features of forest are attributable to the size and shape of the organisms alone (i.e., independent of the species of which they are composed).

In the octocoral forests of St. John, conservation of the colony size-frequency distributions, despite variation in species composition, suggests species redundancy plays a role in maintaining the vertical structure of the community. Furthermore, the variation over time in the relative abundances of species in the larger size classes (Fig. 6) is suggestive of response variation and portfolio effects. Changes in the < 20 cm size class over the course of the study reflect differential mortality to small colonies as well as recruitment, which also varied over the course of the study^74^. The capacity of large (i.e., tall) octocoral colonies to form a cohesive canopy is an important community-scale feature of octocoral forests^75,76^, and it is positively associated with octocoral recruitment on shallow (< 9 m depth)^77^ and mesophotic^78^ reefs. Further, on shallow Bahamian reefs, recruitment and adult abundance of *Antillogorgia elisabethae* were positively associated^79^, and evidence of cause-and-effect in such relationships has been provided^78^. In a manipulative experiment conducted on a tropical mesophotic reef^78^, invertebrate recruitment increased around octocoral colonies (versus bare substratum), with this outcome attributed to hydrodynamic effects associated with the arborescent structure of octocoral colonies, and biotic effects attributed to the role of live octocorals (versus their dead skeletons). Together, the aforementioned studies support two conclusions. First, that octocoral canopies promote community resilience through hydrodynamically-mediated enhancement of recruitment in the understory habitat. Second, that octocoral species capable of producing colonies of comparable size, especially those that are tall and define “canopy species” (sensu Clark and Clark^80^), may be functionally redundant in so far as canopy formation that favors community resilience.

Analyses of octocoral sizes in St. John (Fig. 5), revealed similarity of size-frequency distributions over time at each site, and small differences following Hurricanes Irma and Maria in 2017. The declines in colony density following the hurricanes (e.g., Fig. 1) was caused by the loss of colonies < 30 cm tall through detachment of their holdfasts from the benthos (see also^25,81^). Analyses of flow regime in the understory habitat suggests that the hydrodynamic effects of the canopy may occur in a threshold manner as a function of octocoral density, with distinctive canopy effects on understory flow regimes only emerging at >  ~ 12 colonies m^-2^ (L. Bramanti unpublished data). Because of the reduced height and low biomass of small colonies, their loss from the benthos is unlikely to have strongly influenced overall community function, for example, by affecting canopy formation or community metabolism. Given the nascent understanding of the effects of octocoral canopies on the flow regime in the understory habitat^62^, and how this, in turn, modulates octocoral recruitment^77^, much remains to be done to understand the mechanistic consequences of canopy formation by octocoral forests.

## Conclusion

Our research in St. John over the last decade has focused on changes in benthic communities favoring dominance by octocorals, and we have started to address the processes facilitating this transition and providing ecological stability to octocoral forests once they have formed^51,60^. Portfolio effects and functional redundancy may be important in this regard, and while evidence remains sparse, our data are consistent with the presence of portfolio effects in this system. Rigorous testing of these effects will require a more complete knowledge of the functional biology of octocorals, and in this regard, progress with scleractinians provides an effective model for how such studies might proceed^82^.

## Supplementary Information

Supplementary Information.

## Data Availability

Data used in the study are available through the Biological and Chemical Oceanography Data Management Office (BCO-DMO), https://www.bco-dmo.org/dataset/682966/data; https://doi.org/10.1575/1912/bco-dmo.751176.1; https://doi.org/10.1575/1912/bco-dmo.765328.1; and https://www.bco-dmo.org/dataset/793259.
