# Supplementary material for: Portfolio effects and functional redundancy contribute to the maintenance of octocoral forests on Caribbean reefs
Source: Sci Rep. 2022 May 2;12:7106. doi: 10.1038/s41598-022-10478-4 (PMC9061744; doi:10.1038/s41598-022-10478-4)

SUPPLEMENTARY INFORMATION

Portfolio effects and functional redundancy contribute to the maintenance of octocoral forests on Caribbean reefs

Edmunds, P.J., Lasker H.R.

Fig. 1. Variation in diversity of octocoral colony size at Grootpan Bay and Europa Bay, as expressed as H' and evenness. Sampling took place in the summers of each year with an additional sampling in November 2017 (i.e., 2017.11)

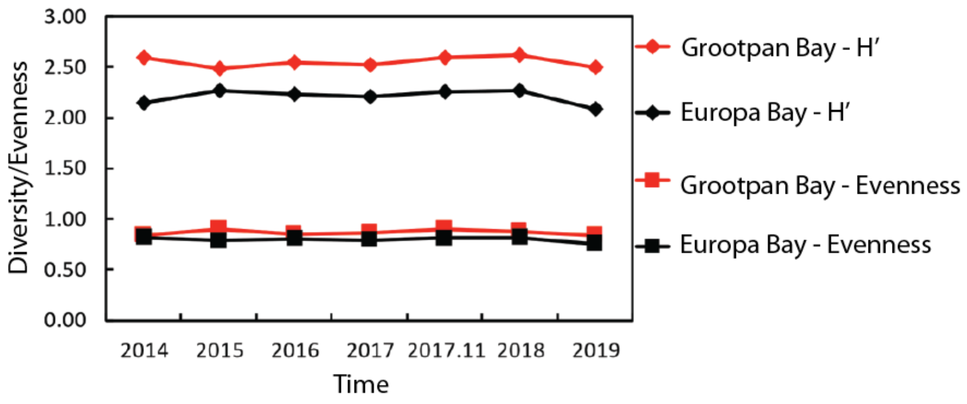

Supplement: Supplementary file 1 — Supplementary Information. [file 41598_2022_10478_MOESM1_ESM.pdf]
